# The Considerations and Controversies in Using High-Flow Nasal Oxygen with Self-Prone Positioning in SARS-CoV-2 COVID-19 Disease

**DOI:** 10.1155/2021/5541298

**Published:** 2021-05-24

**Authors:** Kieran P. Nunn, Murray J. Blackstock, Ryan Ellis, Gauhar Sheikh, Alastair Morgan, Jonathan K. J. Rhodes

**Affiliations:** ^1^Intensive Care Unit, Western General Hospital, Crewe Road South, Edinburgh EH4 2XU, UK; ^2^Intensive Care Unit, Royal Infirmary of Edinburgh, 51 Little France Crescent, Edinburgh EH16 4SA, UK; ^3^Edinburgh Critical Care Research Group (ECCRG), The University of Edinburgh, The Royal Infirmary of Edinburgh, 51 Little France Cres., Edinburgh EH16 4SA, UK

## Abstract

Evidence exists for the use of high-flow nasal oxygen (HFNO) in the general critical care population for acute hypoxemic respiratory failure. There is discord between guidelines for hypoxemia management in COVID-19. Both noninvasive management and intubation present risk to patients and staff and potentially overwhelm hospital mechanical ventilator capacity. The use of HFNO has been particularly controversial in the UK, with oxygen infrastructure failure. We discuss our experience of managing COVID-19 with HFNO and awake self-prone positioning. We focus upon the less-usual case of an eighteen-year-old female to illustrate the type of patient where HFNO may be used when perhaps earlier intubation once was. It is important to consider the wider implications of intubation. We have used HFNO as a bridge to intubation or as definitive management. As we await clinical trial evidence, HFNO with self-prone positioning has a role in COVID-19 for certain patients. Response parameters must be set and reviewed, oxygen infrastructure considered, and potential staff droplet exposure minimised.

## 1. Introduction

Pandemic SARS-CoV-2 infection's most immediately life-threatening feature is the novel interstitial lung disease (COVID-19) causing hypoxemic respiratory failure due to viral pneumonitis and ARDS (Acute Respiratory Distress Syndrome). Health systems are pressurised in preparing for anticipated surges in patient numbers requiring respiratory support. There were early concerns about ventilator availability. We may face this again. Patients present with variable, fluctuating hypoxemic respiratory failure, often in the absence of dyspnoea. The pathophysiology of dyspnoea is complex, involving multiple chemo- and mechanoreception afferents. So, the absence of dyspnoea does not necessarily mean that hypoxemia is benign. Significant uncertainty exists over the optimal therapeutic target when correcting hypoxemia in COVID-19 respiratory failure. Tolerable levels of hypoxemia versus concern over pulmonary oxygen toxicity in acute lung injury must also be balanced against local logistical factors concerning the means to deliver different types of supplemental oxygen therapy safely and the infrastructure these require. In a trial stopped for safety after 205 patients were enrolled, it emerged that conservative oxygen targets in the early management phase of ARDS, PaO_2_ 55 mmHg–70 mmHg (oxygen saturation (SpO_2_) 88-92%), compared to liberal PaO_2_ 90 mmHg–105 mmHg (SpO_2_ > 95%), could be harmful [1]. The Surviving Sepsis group recommend targeting SpO_2_ 92%-96% in adults with COVID-19 [2, 3].

The decision-making when treating hypoxemia is complex and controversial in COVID-19 for many reasons. Recently in the UK, we have seen hospital oxygen supply infrastructure failures with consequent Incident Reporting & Investigation Centre reports (IM/2020/005) [4] and National Patient Safety Alerts (NatPSA/2020/002/NHSPS) [5]. Noninvasive options for advanced supplemental oxygen delivery, including continuous positive airway pressure (CPAP) and high-flow nasal oxygen (HFNO), are inconsistently recommended. And there are concerns about both variable consumption and excessive use of oxygen in addition to the potential risk of cross-infection to other patients and staff. In the USA, the Society of Critical Care Medicine along with the National Institutes of Health (NIH) [6], the European Society of Intensive Care Medicine, the Australian and New Zealand Intensive Care Society, and the World Health Organisation recommends HFNO with effective infection control measures including personal protective equipment (PPE) when supplemental oxygen via nasal canulae or simple facemask systems are ineffective and immediate intubation is not required [2, 7–10]. In the UK, the Faculty of Intensive Care Medicine, the Intensive Care Society, the Association of Anaesthetists of Great Britain and Ireland, and the Royal College of Anaesthetists do not support the use of HFNO or similar devices [11]. A lot of this COVID-19-specific guidance is understandably based on early information and experience (Table 1). Because of this, these organisations have established web resources which are frequently updated.

We present an illustrative case of COVID-19 pneumonitis that was managed with HFNO and self-prone positioning. We summarise HFNO in the management of 16 COVID-19 patients admitted to our Intensive Care Unit (ICU) (Table 2). This was in line with our pre-COVID-19 treatment of some mild ARDS patients. A recent network meta-analysis of noninvasive oxygenation strategies and all-cause mortality in non-COVID-19 acute hypoxemic respiratory failure is reassuring of the use of these strategies pre-COVID-19. Yet even before COVID-19, the relative benefits of each strategy were not clear [12]. In considering our management, a systematic literature search was conducted through June 2020 to identify any publication of patients with COVID-19 treated with HFNO. The following electronic bibliographic databases were searched from 1974 until June 16, 2020, using a comprehensive search strategy developed by an information specialist: Embase, Medline, DynaMed, and The Right Decision. We separately searched society, professional body, and government guidelines as well as identified references. The full texts of all articles identified as relevant during title and abstract screening were obtained and reviewed. The comprehensive search strategy is described in the online supplement (available here). As anticipated, it is too early to have trial evidence. But it remains important to discuss experience whilst decisions on oxygen treatment are being made every day.

## 2. Case Presentation

An 18-year-old female presented in the early hours of the morning on day 1. Having previously consulted primary care services, she was confirmed SARS-CoV-2 COVID-19 positive 3 days prior to hospitalisation through community testing. She had followed Scottish COVID-19 lockdown guidance, but she did work directly with the public in a UK supermarket chain that was also taking distancing and protection precautions. She had become short of breath at rest: respiratory rate (RR) 48 breaths per minute and SpO_2_ 85% on air. She described a productive cough and pleuritic chest pain. She also gave a history of intermittent fever, blocked nose and sore throat, bad taste, and reduced appetite. Seventeen hours later, she was admitted to ICU. Her weight had been stable (104 kg BMI 36). She had a history of chronic widespread pain, migraines, fibromyalgia, anxiety, and irritable bowel syndrome. Her medication history was medroxyprogesterone 10 mg, fluoxetine 20 mg, propranolol modified release 80 mg, almotriptan 12.5 mg, and nortriptyline 30 mg. She had never smoked and did not consume alcohol at all. She studied full time at university up until COVID-19 closure and lockdown.

**Figure 1:**
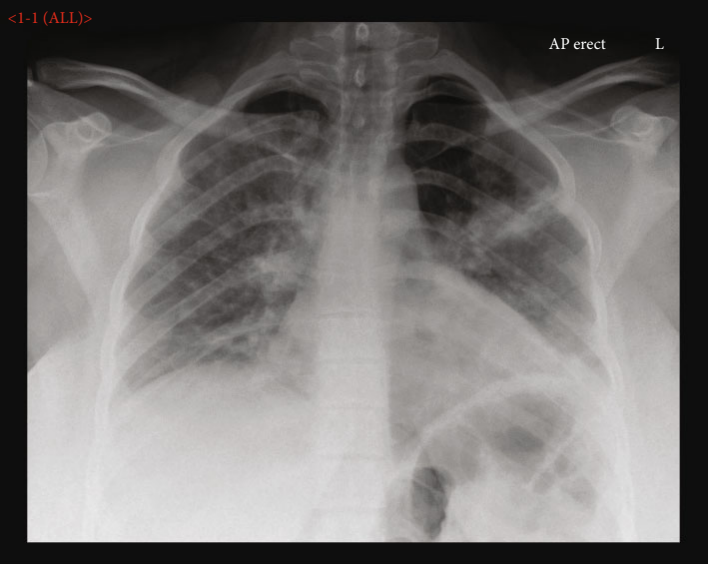
Chest X-ray on admission to the hospital prior to intubation.

**Figure 2:**
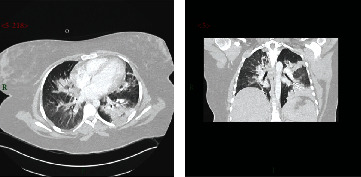
CTPA demonstrated COVID-19 pneumonitis throughout and left consolidation. Appearances in the left lung may suggest superadded bacterial consolidation on a background of bilateral COVID-19 pneumonitis. Appearances in the right lung are more suggestive of COVID-19 pneumonitis.

Initial ward management included supplemental oxygen fraction (FiO_2_) 35% by a fixed performance mask. Her observations at this stage were SpO_2_ 96%, blood pressure (BP) 138/73 mmHg, heart rate (HR) 118/min, and temperature 38.4°C. She reported feeling better, but she looked unwell from the end of the bed, appearing pale, clammy, and tachypnoeic. Her peripheries were warm with normal heart sounds and no oedema; calves were soft and nontender. She remained unable to complete most sentences. Chest X-ray showed bilateral peripheral nodular opacification, most marked in the left mid zone, in keeping with consolidation (Figure 1). The arterial blood gas (ABG) confirmed hypoxaemia PaO_2_ 75 mmHg (P:F 215 mmHg), PaCO_2_ 29 mmHg, H^+^ 34 nmol/L, and base excess -2.8 mmol/L. A prompt referral to critical care was made, and when that consult occurred, SpO_2_ was maintained at 94-96% with FiO_2_ 40% and the temperature was 38.4°C. C-reactive protein (CRP) was 163 mg/L, and full blood count, liver function and urea, and electrolytes were normal. She had received intravenous amoxicillin. After initial improvement however, she was admitted to our mixed level 2/3 critical care having deteriorated to SpO_2_ 96% on FiO_2_ 60% by a fixed performance mask and RR 35/min unstressed but 38°C with mean arterial pressure (MAP) 60 mmHg. On route to the ICU, a CT pulmonary angiogram (CTPA) was performed (Figure 2). This excluded a pulmonary embolism and confirmed COVID-19 changes with left upper lobe consolidation. On admission into a single occupancy room, an arterial line was sited, intravenous crystalloid in targeted boluses used, HFNO started (Optiflow+ (Fisher & Paykel Healthcare), Courtaboeuf, France; heat and moisture exchanger (HME) filter; MR850 heater humidification device and MR290 autofill chamber (Fisher & Paykel Healthcare); and an air/oxygen blender capable of 100% O_2_ and up to 70 L/min flow (Bio-Med Devices, Guilford, CT, USA)), and 0.1 mg/kg oral morphine given with encouragement to self-prone. PaO_2_ was 45 mmHg on 15 L/min O_2_ (mask) and improved over the first period in intensive care on 60% FiO_2_ at 60 L/min HFNO with PaO_2_ 65 mmHg, PaCO_2_ 31 mmHg, SpO_2_ 94%, MAP 77 mmHg, and HR 120/min.

We aimed for SpO_2_ > 92%, PaO_2_ equal or over 60 mmHg, and mean arterial pressure 65 mmHg. Self-prone positioning was encouraged when the P:F ratio was under 150 mmHg (20 kPa). Clarithromycin was added to amoxicillin. Prophylactic low-molecular weight heparin (LMWH) was prescribed with LMWH (anti-Xa) assay monitoring, aiming for 0.1–0.4 *μ*/mL. We asked her to lie on her front for as long as possible. She was able to tolerate this most of the time, only turning supine for care, meals, and interventions. We did not stipulate a maximum prone time as the advantage of being awake is that patients can self-adjust pressure areas as well as engage more fully with physiotherapy and nursing intervention.

**Figure 3:**
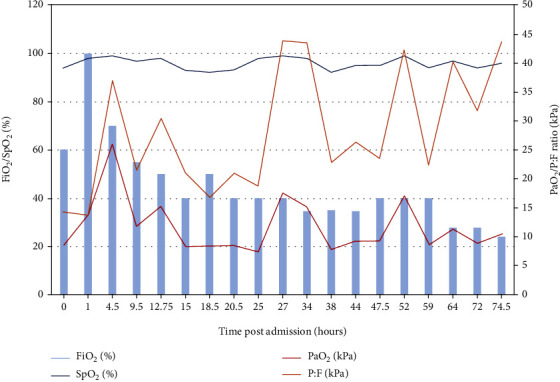
The patient trend in FiO_2_, SpO_2_, PaO_2_, and P:F ratio throughout her time in critical care (P:F 40 kPa = 300 mmHg, P:F 26.7 kPa = 200 mmHg, and P:F 13.3 kPa = 100 mmHg). Self-prone positioning was encouraged when the P:F ratio was under 150 mmHg (20 kPa). We did not stipulate a maximum prone time; this was determined by patient comfort, tolerance, and between-care episodes requiring the patient to be supine.

On day 2, the HFNO was titrated between 40 and 70 L/min. The trend in FiO_2_, SpO_2_, PaO_2_, and P:F ratio throughout her time in critical care shows improvement and fluctuation with time self-prone (Figure 3). Typically, her observations were stable at RR 34/min, SpO_2_ 95%, PaO_2_ 157 mmHg, and PaCO_2_ 35 mmHg with self-prone positioning and her PaO_2_ was under 60 mmHg when supine. She would desaturate to a low SpO_2_ 80%-88% range on movement for nursing care. The use of the broad-spectrum antimicrobial cover was continued in line with daily microbiologist input. On day 3, the HFNO support was reduced to 40–50 L/min, FiO_2_ 40%, RR 22/min, and SpO_2_ 91%. Remaining prone (P:F ratio 140 mmHg, PaO_2_ 56 mmHg), she was comfortable, afebrile, RR 18-24/min, alert, and orientated. Her heart rate had settled to 67 bpm and BP 114/65. She continued to self-prone through day 4. Part of the success of this strategy was to ensure her comfort with measures such as extra pillows under pressure points of her pelvis. She remained in positive spirits, and overall, she found the patient interface of HFNO acceptable and did not like the idea of CPAP or invasive ventilation. On day 5, she completed co-amoxiclav, HFNO flow was reduced to a minimum, and FiO_2_ was 40%; eventually, she was liberated from HFNO onto a 24-28% fixed performance oxygen mask. On day 6, she was discharged back to infectious diseases having not been intubated. She reported feeling physically much better but remained anxious and found it difficult to process the events of the last few days. She remained tired and lethargic but subjectively less short of breath, and all chest pain had resolved. By day 7, tiredness and the cough were the main complaints but the cough was now nonproductive. Assistance was required when walking to the toilet. Mobility gradually improved. Psychiatry was consulted and felt that she seemed to have coped well with being in the hospital, including ICU, but had raised concern about remembering any new detail that may become hard to process pertaining to this spell of hospitalisation and ill health. Strategies for managing this were provided. On day 9, with RR 19/min, SpO_2_ 98% on room air, BP 101/67 mmHg, HR 104/min, temperature 36.5°C, and feeling well, she was discharged home.

## 3. Discussion

We were initially deterred from our usual practice with HFNO in hypoxemic type 1 respiratory failure by the early rhetoric in COVID-19. Prior to COVID-19, in hypoxemic type 1 respiratory failure, we used HFNO for some patients as a bridge to intubation or as definitive treatment where response and sustained improvement was demonstrated. It remains important not to delay inevitable intubation. We intubated 75% of COVID-19 patients admitted to ITU which dropped to 57% when we started using HFNO. The relatively high COVID-19 mortality in our HFNO group (31%) compared to our overall COVID-19 mortality (20%) comes from a relatively small sample from which general inferences cannot be made. Yet, it is important to acknowledge that this mortality was made up from patients who went on to be intubated and then died or where an active decision not to intubate had been made.

The case discussed highlights the importance of accounting for patient, hospital, and staff safety factors and of considering patient demographics and history, including psychiatric history in the decision between NIV, including HFNO, and intubation. We know that the ITU psychological burden is especially high with intubation and sedation [13, 14]. We fortuitously have not had any staff testing COVID-19 positive, and we are deeply saddened by the morbidity and mortality amongst healthcare workers worldwide. Notwithstanding, this is in accord with the prior opinion that aerosolization from HFNO may be insignificant when infection control practice and PPE are optimal [15–18].

All currently available guidelines were considered for an individualised patient approach. In some cases, HFNO allowed time for the appropriateness of escalation to invasive ventilation to be determined as being in the patients' best interest. This is a crucial bridging role of HFNO. We have found that HFNO is a comfortable, tolerable patient interface in both the supine and prone positions and has not had compliance issues allowing constant usage. COVID-19 pneumonitis causes a heterogenous lung due to ventilation-perfusion ratio (*V*˙/*Q*˙) mismatching, a significant shunt fraction, and contributing lung microthrombi causing impaired hypoxic pulmonary vasoconstriction [19]. Prone positioning is beneficial in invasively ventilated ARDS patients, and there is emerging evidence for awake self-prone positioning [19, 20]. When we have used CPAP by the mask interface, compliance has been an issue in some cases. This has previously been reported [21–23]. A mask limits head position in a self-prone strategy. HFNO device tolerance and thus, by implication, better compliance and reduced frustration-led patient interface disconnections are also likely to confer relative healthcare worker protection. Prior to COVID-19, it was not our usual practice to use CPAP in this setting and this may have influenced our perception. Similar cases have started to be reported [24–31]. HFNO cannot change the natural course of viral pneumonia, but neither can intubation, and CPAP/NIV has not previously been recommended [32]. In patients not responding to supplemental oxygen with improved PaO_2_, there is more likely to be a significant intrapulmonary shunt and invasive ventilation is probably required imminently, but in supplemental oxygen responders, the pathophysiology is predominantly ventilation-perfusion mismatching. Arterial oxygenation can be safely sustained with oxygen therapy in this latter group [33].

The early National Institute for Health and Care Excellence (NICE) COVID-19 rapid guideline advised that when an adult is admitted to hospital, an early assessment of frailty is made which can be either individualised or by the Clinical Frailty Scale score [34] as part of determining disposition.

HFNO is established in critical care [12, 35, 36]. Combination therapy in ARDS with prone positioning is described [37]. FLORALI publication in 2015 made it clear that intubation should not be delayed [12, 38]. Persistence with noninvasive methods can result in patient self-inflicted lung injury [7, 19, 39]. A key question from the FLORALI papers was whether HFNO could prevent intubation. However, it is important to consider that most patients in this trial had pneumonia and did not all meet the current criteria for ARDS. Therefore, the results may not be generalisable to all patients with hypoxemic respiratory failure. Yet this study did suggest that HFNO is noninferior to other oxygen delivery methods including noninvasive ventilation (NIV/CPAP) in reducing the subsequent need for intubation. HFNO reduced ICU and 90-day mortality compared with other strategies. It also subjectively improved dyspnoea and respiratory discomfort, important for patient experience, at 1 hour compared with the other oxygen delivery devices [38].

The UK National Health Service (NHS) has published COVID-19 guidance [40, 41]. It is recommended that NIV (including HFNO) is delivered in a negative pressure room with air exchanges greater than 10 cycles per hour or in an air-cycled neutral pressure room or side room. In anticipation of the first COVID-19 patients being admitted, our unit was sealed and modified for full droplet precaution. We use filtering facepiece 3 (FFP3) plus visor and fluid-resistant gown PPE and have greater than 50% of beds in single occupancy rooms.

**Figure 4:**
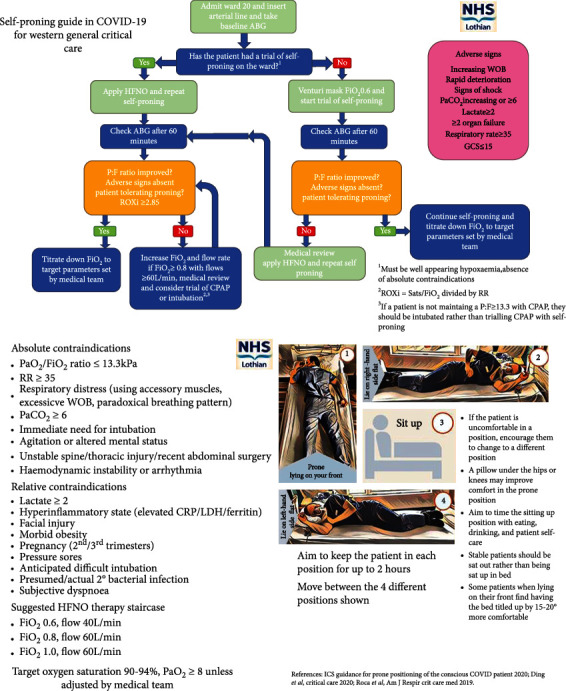
Western General Hospital, Edinburgh, NHS Lothian, NHS Scotland. Awake self-prone guideline.

Where there is no adequate response, where clinical decline continues, or where patient tolerance limits use, early intubation and mechanical ventilation should be pursued where appropriate. In COVID-19 with full FFP3 PPE, human factor challenges are compounded by staff training challenges (redeployment in the pandemic), fatigue, and the visual and hearing cue limitations associated with masks and visors. This amalgamates to make ICU intubation more difficult and possibly more hazardous than usual. Where intubation is not needed, it can be argued that consequently the clinician is protected from intubation risks and we can consider the resource-saving benefit to the broader population. The ROX index may play a predictive role in identifying failing HFNO and need for intubation. Whilst it lacks external validity, it utilises three bedside observations (SpO_2_, FiO_2_, and RR) to standardise the degree of hypoxic respiratory failure [42–44]. We consider this to be additionally helpful in our local guidance for trialling self-prone positioning in critical care (Figure 4), based on Intensive Care Society guidance [45].

We have not used HFNO outside of critical care. This helped both in the monitoring of total hospital oxygen usage and in the advocated approach of using it as a targeted, duration-limited therapy with a clear escalation or deescalation exit strategy based on both clinical judgement and understanding of oxygen infrastructure. HFNO should be confined to use where regular review and goal setting are possible. Consideration needs to be given to current patient numbers on oxygen in the hospital and the hospital infrastructure including the capacity of the Vacuum Insulated Evaporator (VIE) and cylinder manifolds as well as the hospital pipework flow capacity. In an audit of our own unit's oxygen consumption, we found marked differences between invasively ventilated patients and those on HFNO, FiO_2_ 0.43 (IQR 0.41-0.45) vs. 0.55 (IQR 0.48-0.60) (*p* = 0.029) for invasive ventilation vs. HFNO, respectively, and fresh oxygen flow rates of 3.0 (IQR 2.6-3.2) vs. 18.4 (IQR 14.4-25.9) L/min (*p* = 0.029) for invasive ventilation vs. HFNO, respectively. Clearly, the use of HFNO has significant resource implications and may not be deliverable in all infrastructures. Indeed, the latent heat of vaporisation of oxygen to convert from liquid to gas through the superheater takes atmospheric energy to change the oxygen phase, with the air-water vapour losing so much energy that it condenses and in excess freezes. Under normal operating conditions, the VIE utilises this latent heat to keep cool, but if usage is high, the temperature in the evaporator will fall too much so that the regulators governing the pipeline pressure will no longer function correctly. The excess demand due to overwhelming case numbers in the COVID-19 pandemic has led to reports of hospital oxygen systems freezing and failing [4, 46, 47].

Organised trials are now needed. RECOVERY-RS (ISRCTN16912075) will look at three different approaches to providing ventilatory support, including HFNO, to patients suspected or confirmed COVID-19. Hopefully, many of the uncertainties of how best to provide noninvasive respiratory support will be answered in a relatively homogeneous population, at least compared with previous studies in ARDS.

## 4. Conclusions

A one-size-fits-all approach to hypoxemic respiratory failure is not possible. We provide data to show that HFNO has a role in selected units and patients alongside real-time oxygen infrastructure consideration. HFNO is possibly suited to and more efficacious with self-prone positioning. A clear plan must be in place to determine the threshold for the failure of HFNO and escalation to intubation and invasive mechanical ventilation, if appropriate. In deciding not to use HFNO due to potential aerosolization concerns, the clinician is committing to alternative therapies that have their own associated risks including aerosolization [48–50].

## Figures and Tables

**Table 1:** A comparison of major organisation recommendations for CPAP/NIV and HFNO.

Body	Noninvasive ventilation	High-flow nasal oxygen
SCCM (USA) [6]	Consider if HFNO failed	Yes
NIH (USA) [6]	Consider if HFNO unavailable	Yes
ANZICS (Australia & New Zealand) [8–10]	No	Yes
WHO (Switzerland) [7]	Yes	Yes
FICM, RCoA, ICS, & AAGBI (UK) [11]	Bridge to intubation	No
ESICM (Europe/UK) [2]	Consider if HFNO failed	Yes
Surviving Sepsis Campaign [2]	Consider if HFNO failed	Yes
NHS England [40]	Yes	No

SCCM: Society of Critical Care Medicine; NIH: National Institutes of Health; ANZICS: Australian and New Zealand Intensive Care Society; WHO: World Health Organisation; FICM: Faculty of Intensive Care Medicine; RCoA: Royal College of Anaesthetists; ICS: Intensive Care Society; AAGBI: Association of Anaesthetists of Great Britain and Ireland; ESICM: European Society of Intensive Care Medicine.

**Table 2:** A case series of our patients treated with high-flow nasal oxygen during the first wave of the COVID-19 pandemic.

Age (years)	Gender	Comorbidities	ICU admission P:F (mmHg)	Initial HFNO P:F (mmHg)	Intubated	Preintubation P:F on HFNO (mmHg)	Proned (i = intubated)	Preprone P:F (mmHg)	Postprone P:F (mmHg)	Preextubation P:F (mmHg)	Outcome
63	M	HTN, BMI, DM	96	113 (FiO_2_ 0.6, flow 40 L/min)	Y	57 (FiO_2_ 1.0, flow 50 L/min)	Y (i)	98 (FiO_2_ 0.7)	222 (FiO_2_ 0.45)	Tracheostomy	I
44	M	BMI, DM	66	93 (FiO_2_ 1.0, flow 60 L/min)	Y	50 (FiO_2_ 1.0, flow 60 L/min)	Y (i)	101 (FiO_2_ 0.55)	140 (FiO_2_ 0.45)	187 (FiO_2_ 0.4)	D/C
60	M		61	71 (FiO_2_ 0.9, flow 65 L/min)	Y	60 (FiO_2_ 1.0, flow 60 L/min)	Y (i)	128 (FiO_2_ 0.5)	88 (FiO_2_ 0.75)	N/A	Died
68	M	HTN, BMI, PAF, CMT	183	165 (FiO_2_ 0.6, flow 60 L/min)	Y	135 (FiO_2_ 0.55, flow 50 L/min)	Y (i)	113 (FiO_2_ 0.9)	285 (FiO_2_ 0.55)	N/A	Died
66	M	TIA, HT	68	59 (FiO_2_ 1.0, flow 60 L/min)	Y	N/A (immediate progression)	Y (i)	N/A (immediate progression)	135 (FiO_2_ 0.55)	N/A	Died
66	M	DM, A	116	116 (FiO_2_ 0.5, flow 60 L/min)	N	N/A	Y	90 (FiO_2_ 0.5)	103 (FiO_2_ 0.6)	N/A	D/C
65	M	HTN, BMI, DM	105	144 (FiO_2_ 0.9, flow 50 L/min)	N	N/A	Y	105 (FiO_2_ 0.6)	104 (FiO_2_ 0.8)	N/A	D/C
67	M	NHL-R	112	116 (FiO_2_ 0.65, flow 40 L/min)	Y	143 (FiO_2_ 60%, flow 40 L/min)	N	N/A	N/A	N/A	Died
53	F	HTN, BMI, DM, S, RA-R	81	126 (FiO_2_ 0.60, flow 50 L/min)	N	N/A (immediate progression)	Y	Not available	Not available	187 (FiO_2_ 0.35)	D/C
47	M	DM, GPA-R	77	65 (FiO_2_ 0.8, flow 60 L/min)	Y	67 (FiO_2_ 0.8, flow 50 L/min)	Y (i)	11	83 (FiO_2_ 0.9)	Tracheostomy	D/C
53	M		99	117 (FiO_2_ 0.6, flow 50 L/min)	N	N/A	Y	Not available	Not available	N/A	D/C
45	M	BMI, G	90	104 (FiO_2_ 0.7, flow 50 L/min)	Y	62 (FiO_2_ 0.9, flow 60 L/min)	N	N/A	N/A	210 (FiO_2_ 0.35)	D/C
54	M	AML	54	HFNC applied for palliation	N	N/A	N	N/A	N/A	N/A	Died
30	M	BMI, A	150	101 (FiO_2_ 0.55, flow 50 L/min)	Y	49 (FiO_2_ 0.75, flow 45 L/min)	Y (i)	105 (FiO_2_ 0.5)	128 (FiO_2_ 0.55)	Tracheostomy	D/C
18	F	BMI, F, D	107	108 (FiO_2_ 0.6, flow 60 L/min)	N	N/A	Y	161 (FiO_2_ 0.7)	329 (FiO_2_ 0.4)	N/A	D/C
51	M	A	141	95 (FiO_2_ 0.8, flow 60 L/min)	N	N/A	Y	Not available	Not available	N/A	D/C

Legend: HTN: hypertension; BMI: BMI > 25; DM: type 2 diabetes; A: asthma; S: smoker; PAF: paroxysmal atrial fibrillation; CMT: Charcot-Marie-Tooth; TIA: transient ischaemic attack; HT: hypothyroid; NHL-R: non-Hodgkin lymphoma-taking rituximab; RA-R: rheumatoid arthritis-taking rituximab; GPA-R: granulomatosis with polyangiitis-taking rituximab; G: gout; AML: acute myeloid leukaemia; F: fibromyalgia; D: depression. Our preference and new admissions were treated with HFNO using the Optiflow+ setup detailed in the case report. If there was a demand on equipment or the patient was later in the phase of weaning or being extubated to HFNO, then we did also deliver HFNO by the Dräger Evita® Infinity® V500 ventilator. I = inpatient; D/C = discharged home; died = death in intensive care.

## Data Availability

Data are available on request from the corresponding author.

## Supplementary Materials

Supplementary Materials The electronic bibliographic database search strategy.
