# Supplementary material for: The Considerations and Controversies in Using High-Flow Nasal Oxygen with Self-Prone Positioning in SARS-CoV-2 COVID-19 Disease
Source: Case Rep Crit Care. 2021 May 24;2021:5541298. doi: 10.1155/2021/5541298 (PMC8142809; doi:10.1155/2021/5541298)
Supplement: Supplementary Materials — The electronic bibliographic database search strategy. [file 5541298.f1.docx]

**Search Strategy**

Medline and Embase were searched in the exact same way. Dynamed is a different kind of database, it contains topic reviews for conditions so on Dynamed search for the condition only. The Right Decision Platform has grouped its COVID resources together to make it easier for swift access to the information and adds to that collection as guidelines, etc become available. Search includes all types of material and does not exclude letters or editorials.

Database: Embase <1974 to 2020 June 16>

Search Strategy:

--------------------------------------------------------------------------------

1 exp Coronavirinae/ (14559)

2 exp Coronavirus infection/ (14269)

3 exp Coronavirus/ or exp Human coronavirus OC43/ or exp SARS coronavirus/ (14559)

4 exp Human coronavirus NL63/ (211)

5 exp Human coronavirus 229E/ (179)

6 (HKU1 or ncov).mp. [mp=title, abstract, heading word, drug trade name, original title, device manufacturer, drug manufacturer, device trade name, keyword, floating subheading word, candidate term word] (1187)

7 1 or 2 or 3 or 4 or 5 or 6 (23091)

8 (SARS or SARS-Cov or MERS or MERS-Cov or Middle East Respiratory Syndrome or camel* or dromedar* or equine or coronary or coronal or covidence ir covidien or influenza virus or HIV or bovine or calves or TGEV or feline or porcine or BCOV or PED or PEDV or PDCOV or FIPV or FCOV or SADS-COV or canine or Ccov or zoonotic or avian influenza or H1N1 or H5N1 or H5N6 or IBV or murine corona*).mp. [mp=title, abstract, heading word, drug trade name, original title, device manufacturer, drug manufacturer, device trade name, keyword, floating subheading word, candidate term word] (1747398)

9 7 not 8 (6351)

10 exp pneumonia/ (295767)

11 (10 or covid*.mp. or coronavirus*.mp. or corona virus*.mp. or ncov.mp. or 2019-ncov.mp. or SARS*.mp.) and Wuhan.mp. [mp=title, abstract, heading word, drug trade name, original title, device manufacturer, drug manufacturer, device trade name, keyword, floating subheading word, candidate term word] (1627)

12 (((2019-ncov or ncov19 or ncov-19 or 2019 novel cov or SARS-cov2 or SARS-cov-2 or SARScov-2 or SARS-coronavirus-2 SARS-LIKE coronavirus orcoronavirus-19 or Covid-19 or covid19 or covid 2019 or (novel or new or nouveau)) adj1 cov*) or ncov or covid or coronavirus* or corona virus or Pandemi* or covid-19 or covid19).mp. [mp=title, abstract, heading word, drug trade name, original title, device manufacturer, drug manufacturer, device trade name, keyword, floating subheading word, candidate term word] (72623)

13 11 or 12 (72658)

14 9 or 13 (74701)

15 (high adj3 oxygen).ti,ab. (10926)

16 14 and 15 (38)

17 from 16 keep 3,5-12,14-20,23-27 (21)

Database: Medline <1974 to 2020 June 16>

Search Strategy:

--------------------------------------------------------------------------------

1 exp Coronavirinae/ (14559)

2 exp Coronavirus infection/ (14269)

3 exp Coronavirus/ or exp Human coronavirus OC43/ or exp SARS coronavirus/ (14559)

4 exp Human coronavirus NL63/ (211)

5 exp Human coronavirus 229E/ (179)

6 (HKU1 or ncov).mp. [mp=title, abstract, heading word, drug trade name, original title, device manufacturer, drug manufacturer, device trade name, keyword, floating subheading word, candidate term word] (1187)

7 1 or 2 or 3 or 4 or 5 or 6 (23091)

8 (SARS or SARS-Cov or MERS or MERS-Cov or Middle East Respiratory Syndrome or camel* or dromedar* or equine or coronary or coronal or covidence ir covidien or influenza virus or HIV or bovine or calves or TGEV or feline or porcine or BCOV or PED or PEDV or PDCOV or FIPV or FCOV or SADS-COV or canine or Ccov or zoonotic or avian influenza or H1N1 or H5N1 or H5N6 or IBV or murine corona*).mp. [mp=title, abstract, heading word, drug trade name, original title, device manufacturer, drug manufacturer, device trade name, keyword, floating subheading word, candidate term word] (1747398)

9 7 not 8 (6351)

10 exp pneumonia/ (295767)

11 (10 or covid*.mp. or coronavirus*.mp. or corona virus*.mp. or ncov.mp. or 2019-ncov.mp. or SARS*.mp.) and Wuhan.mp. [mp=title, abstract, heading word, drug trade name, original title, device manufacturer, drug manufacturer, device trade name, keyword, floating subheading word, candidate term word] (1627)

12 (((2019-ncov or ncov19 or ncov-19 or 2019 novel cov or SARS-cov2 or SARS-cov-2 or SARScov-2 or SARS-coronavirus-2 SARS-LIKE coronavirus orcoronavirus-19 or Covid-19 or covid19 or covid 2019 or (novel or new or nouveau)) adj1 cov*) or ncov or covid or coronavirus* or corona virus or Pandemi* or covid-19 or covid19).mp. [mp=title, abstract, heading word, drug trade name, original title, device manufacturer, drug manufacturer, device trade name, keyword, floating subheading word, candidate term word] (72623)

13 11 or 12 (72658)

14 9 or 13 (74701)

15 (high adj3 oxygen).ti,ab. (10926)

16 14 and 15 (38)

17 from 16 keep 3,5-12,14-20,23-27 (21)
